# Santorinicele without pancreas divisum pathophysiology: initial clinical and radiographic investigations

**DOI:** 10.1186/1471-230X-13-62

**Published:** 2013-04-09

**Authors:** Wataru Gonoi, Hiroyuki Akai, Kazuchika Hagiwara, Masaaki Akahane, Naoto Hayashi, Eriko Maeda, Takeharu Yoshikawa, Shigeru Kiryu, Minoru Tada, Kansei Uno, Naoki Okura, Kazuhiko Koike, Kuni Ohtomo

**Affiliations:** 1 Department of Radiology, Graduate School of Medicine, The University of Tokyo, Bunkyo-ku, Tokyo, Japan; 2 Department of Computational Diagnostic Radiology and Preventive Medicine, The University of Tokyo Hospital, Bunkyo-ku, Tokyo, Japan; 3 Department of Radiology, The Institute of Medical Science, The University of Tokyo, Minato-ku, Tokyo, Japan; 4 Department of Gastroenterology, Graduate School of Medicine, The University of Tokyo, Bunkyo-ku, Tokyo, Japan; 5 Department of Radiology, Japan Labour Health and Welfare Organization, Kanto Rosai Hospital, Kawasaki City, Kanagawa, Japan

**Keywords:** Congenital anomaly, Cross-sectional study, Dorsal pancreatic duct, Magnetic resonance imaging, Pancreatic duct, Wirsungocele

## Abstract

**Background:**

Only one case of santorinicele without pancreas divisum pathophysiology (SWOPP) was previously reported. The purpose of the study was to determine the gross prevalence of SWOPP and santorinicele with pancreas divisum (SWPD) in community and patient populations, and investigate their clinical and radiographic features.

**Methods:**

This cross-sectional study was performed at a tertiary referral centre. The Patient group comprised 2035 consecutive patients enrolled in the study who underwent magnetic resonance cholangiopancreatography (MRCP) studies. The Community group comprised 2905 consecutive subjects who participated in our whole-body medical check-up program that routinely includes MRCP studies. SWOPP was diagnosed when a saccular dilatation of the terminal portion of the dorsal pancreatic duct was observed unaccompanied by pancreas divisum or dominant dorsal duct. The prevalence of SWOPP and SWPD, and the clinical and radiological features were assessed in each group.

**Results:**

Five cases of SWOPP were found in the Patient group (age range, 67–85 years; mean age, 73.6 years) (5/2035 = 0.25%; 95% confidence interval, 0.07–0.57); there were no cases of SWOPP in the Community group (0/2905 = 0.00%; 95% confidence interval, 0.00–0.10) (P = 0.01). Previous history of pancreatitis (4/5) and chronic pancreatitis (3/5) was more common in patients with SWOPP than in other subjects in the Patient or Community groups (both P < 0.05). Two cases of SWOPP were accompanied by reverse-Z type meandering main pancreatic duct. Six cases of SWPD were found. These cases were asymptomatic in 4/6, had a larger santorinicele (6.9 mm) than SWOPP patients (4.5 mm; P = 0.02), and were not associated with pancreatitis (0/6).

**Conclusions:**

The second to sixth reported cases of SWOPP were presented. SWOPP is a relatively rare condition found mostly in patients suffering pancreatitis, especially chronic pancreatitis, and may be an acquired condition. Santorinicele is not always accompanied by pancreas divisum.

## Background

Santorinicele is a focal cystic dilatation of the terminal portion of the dorsal pancreatic duct, just proximal to the minor papilla, and was first described in 1994 by Eisen et al. [1], who reported four patients with pancreatitis and pancreas divisum accompanied by a focal cystic dilatation of the terminal portion of the dorsal pancreatic duct. Since the first description, all reported santoriniceles have been in patients with pancreas divisum, either complete or incomplete [2-7], and are speculated to result from impeded pancreatic flow [1,6].

A series of studies classified the morphology of dorsal pancreatic duct at its terminal portion into stick, branch, cudgel, spindle, and saccular types [8-10]. Saccular type appears to be compatible with santorinicele, but these reports did not investigate the presence or absence of pancreas divisum, or clinical background such as pancreatitis.

Three cases of santorinicele not accompanied by pancreas divisum were subsequently reported as a new entity: "santorinicele without pancreas divisum" [11-13]; however, the latter two cases [12,13] were accompanied by a dominant dorsal pancreatic duct, in which the calibre of the dorsal pancreatic duct was wider than that of the ventral duct, which is similar pathophysiology to that of pancreas divisum.

To the best of our knowledge, no previous studies have closely investigated the prevalence of santorinicele without pancreas divisum pathophysiology (SWOPP), or evaluated its clinical and radiographic features. The purpose of the present study was to elucidate the clinical and radiographic features of SWOPP, including its gross prevalence in a community population and in a patient population from our hospital, based on the 1.5/3 T high-field magnetic resonance (MR) technique.

## Methods

Approval for the present study was obtained from the Research Ethics Committee of the University of Tokyo Hospital, Japan.

### Subjects

The subjects were classified into two groups: a Patient group and a Community group. The Patient group comprised consecutive patients who underwent abdominal MR scans including magnetic resonance cholangiopancreatography (MRCP) between December 1 2004 and May 31 2010 in our academic tertiary care hospital. The Community group comprised consecutive subjects in a community population who responded to leaflets and Internet advertising, and participated in a whole-body medical check-up program hosted by our hospital between October 12 2006 and May 31 2010. The patients were enrolled cross-sectionally. The program included abdominal MR scans and MRCP; evaluation of smoking habits (Brinkman index, daily number of cigarettes × years), drinking habits (daily alcohol consumption), and medical history; an interview regarding subjective symptoms; and a physical examination by a physician. Written informed consent was acquired prior to enrolment into the study from all subjects in both groups. It included not only agreement to enrolment into the study but also agreement to the publication of some identifying information such as age, gender, disease, family history, clinical and radiographic findings, but excluding the subject’s name and photographs of their face. In the event that a subject in the Community group was sent to our hospital for a detailed examination and found to belong to both groups, he/she was included only in the Community group.

In assessing the history of subjects in the Patient group, subjects were recorded as having acute or chronic pancreatitis as of the diagnosis in January 2012 according to the latest published criteria [14,15], while the history of subjects in Community group was acquired during an interview by a physician; therefore, in this group, the diagnostic criteria for pancreatitis were not fully specified. Possible pancreatic pain was considered when a subject had upper abdominal pain or back pain that was consistent with the pain caused by a pancreatic disease, regardless of the possibility of other disease.

### Imaging studies

In the Patient group, MR studies were performed on 3-tesla scanners (SIGNA EXCITE HDx, GE Healthcare Japan, Tokyo, Japan) or on 1.5-tesla scanners (SIGNA EXCITE HD and HDx, GE Healthcare; MAGNETOM Avanto, Siemens AG, Erlangen Germany; EXCELART, Toshiba Medical Systems, Tochigi, Japan). Acquired sequences were heavily T2-weighted MRCP images using breath-hold two-dimensional half-Fourier fast spin echo (repetition time/echo time = 2400–∞/600–1100 ms; slice thickness = 30–50 mm) and respiratory-gated three-dimensional half-Fourier fast spin echo (repetition time/echo time = 1300–∞/500–900 ms; slice thickness = 1.2–2.0 mm with no gap). Coronal and oblique-coronal projection images were reconstructed for MRCP images. T2-weighted, T1-weighted, and diffusion weighted images were also acquired.

In the Community group, MR studies were performed on a 3-tesla scanner (SIGNA EXCITE HDx, GE Healthcare Japan). Acquired sequences were heavily T2-weighted MRCP images in the coronal plane using breath-hold two-dimensional half-Fourier fast spin echo (repetition time/echo time = ∞/600 ms; slice thickness = 40 mm) with four coronal and oblique-coronal projection images. T2-weighted, T1-weighted, and diffusion weighted images were also acquired.

### Image review

All MR images were interpreted on picture archiving and communication system workstations (Centricity, GE Medical Systems) by two board-certified radiologists with experience in distinguishing pancreatic ductal anatomy, who were not informed of the clinical information. Discrepancies among opinions were settled by discussion.

**Figure 1:**
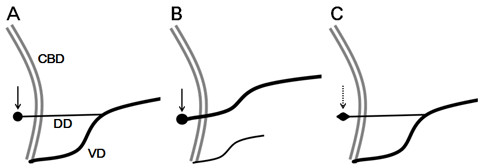
**Santorinicele with and without pancreas divisum.** Santorinicele was defined as a focal saccular dilatation of the terminal end of the dorsal pancreatic duct (solid arrows), and classified into two patterns according to the presence or absence of pancreas divisum: (**A**) santorinicele without pancreas divisum pathophysiology (SWOPP) and (**B**) santorinicele with pancreas divisum (SWPD). (**C**) Dorsal pancreatic duct with spindle-type dilatation (dotted arrow) was not considered as a santorinicele because it has different pathophysiology. CBD, common bile duct; DD, dorsal (pancreatic) duct; VD, ventral (pancreatic) duct.

Santorinicele was defined as a focal saccular dilatation of the terminal portion of the dorsal pancreatic duct, in which the calibre changes steeply, as defined previously (Figure 1) [8-12]. SWOPP was diagnosed when a santorinicele was found unaccompanied by pancreas divisum (complete or incomplete) or by dominant dorsal duct [16]. Subjects having the following findings were excluded: (1) spindle, cudgel, stick, or branch types of the terminal portion of the dorsal pancreatic duct, in which the calibre changes smoothly (Figure 1) [8-10]; (2) neoplasm in the dorsal portion of the head of pancreas; (3) suspected intraductal papillary mucinous neoplasm in the dorsal pancreatic duct; and (4) other major malformation such as abnormal arrangement of pancreaticobiliary ducts or choledochocele. Minor ventral ductal anatomical variation that does not seem to change the flow of pancreatic juice in the dorsal pancreatic duct, such as meandering main pancreatic duct (loop type or reverse-Z type) [17] may exist. Santorinicele with pancreas divisum (SWPD) was diagnosed for a santorinicele accompanied by pancreas divisum (classical type, pancreas divisum with absent ventral duct, incomplete type) [16] using the same exclusion criteria. When a case of SWOPP or SWPD was diagnosed, the clinical and radiographic information was recorded.

The radiologists were first asked to review all the radiographic records of participants for any dorsal pancreatic ductal lesions or pancreatic cystic lesions. All images of the extracted cases were then reviewed and investigated for the presence of santorinicele. When santorinicele was diagnosed, its size was measured along the axis of the dorsal pancreatic duct (horizontal diameter) and in an axis vertical to the horizontal diameter (vertical diameter). Values obtained from the two radiologists were averaged.

### Statistical analysis

Fisher’s exact test was used to compare the prevalence of SWOPP or SWPD on MRCP studies between the Patient and Community groups. Diagnosis of SWOPP or SWPD, and the Patient group were compared in terms of gender, pancreatitis, and chronic pancreatitis, also using Fisher's exact test. Diagnosis of SWOPP or SWPD, the Patient group, and the Community group were compared in terms of age, alcohol consumption, and Brinkman index, using Student's *t*-test. The level of statistical significance was set at 0.05. Family-wise error was corrected by Bonferroni’s correction for each section. All statistical computing was performed using the free software R Ver. 2.9 (The R Foundation for Statistical Computing, Vienna, Austria, http://cran.r-project.org/).

## Results

### Subjects

A total of 4,940 subjects were enrolled; of these, 149 subjects found to belong to both the Patient and Community groups were assigned to the Community group.

Enrolled in the Patient group were 2,035 patients treated at our hospital: 964 females (age, 9–93 years; mean age, 63.7 years) and 1,071 males (age, 7–93 years; mean age, 64.2 years). A history of non-tumour induced pancreatitis and chronic pancreatitis was found in 11.5% (235/2035) and 8.50% (173/2035), respectively, of these patients.

Enrolled in the Community group were 2,905 subjects who participated in the health check program hosted by our hospital: 1,151 females (age, 20–84 years; mean age, 56.2 years) and 1,754 males (age, 23–88 years; mean age, 55.9 years). Mean alcohol consumption was 29.0 g/day (standard deviation, 44.7 g/day) and mean Brinkman index was 314 (standard deviation, 459). A history of pancreatitis and chronic pancreatitis was found in 1.10% (32/2905) and 0.41% (12/2905) respectively, of these subjects.

**Figure 2:**
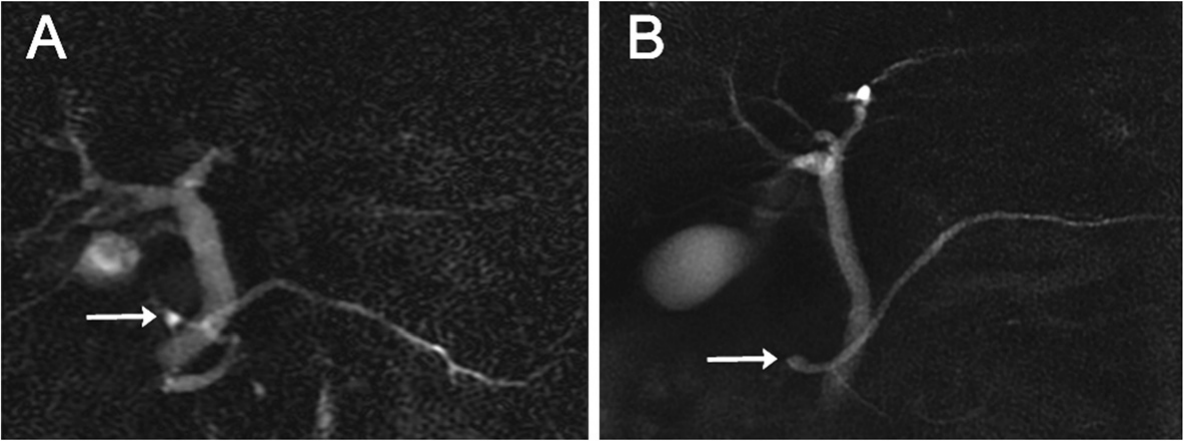
**Santorinicele depicted on magnetic resonance cholangiopancreatography.** Arrows indicate the santorinicele. (**A**) A case of santorinicele without pancreas divisum pathophysiology, defined as a saccular dilatation of the distal dorsal duct just proximal to the minor papilla, unaccompanied by pancreas divisum or dominant dorsal duct (Case 5). (**B**) A case of santorinicele with pancreas divisum, a saccular dilatation of the distal dorsal duct just proximal to the minor papilla and which is accompanied by pancreas divisum (Case 6).

From the imaging studies of all participants, we extracted 1,193 studies that had any type of dorsal pancreatic ductal lesion or pancreatic cystic lesion. Image review of these studies revealed only five cases of SWOPP (age range, 67–85 years; mean age, 73.6 years; females/males, 3/2) (Figure 2). All cases were from the Patient group.

Image review also revealed six patients with SWPD (five from the Patient group and one from the Community group: age range, 56–78 years; mean age, 69.2 years; females/males, 1/5) (Figure 2).

### Clinical and radiographic features of SWOPP

The clinical and radiographic features of the five patients with SWOPP are summarized in Table 1. Four of the five patients (80%) had a history of pancreatitis [three with chronic pancreatitis (60%) and one with acute pancreatitis (20%)], and three of the five (60%) had possible pancreatic pain, including two patients with both a history of pancreatitis and possible pancreatic pain. One patient was asymptomatic (20%). Mean alcohol consumption was 50.0 g/day (range, 0–150 g/day). Mean Brinkman index was 268 (range, 0–920). No case of SWOPP was accompanied by peri-ampullary diverticulum or any major pancreaticobiliary anomaly; however, two of the five cases were accompanied by the ventral ductal anatomical variation of reverse-Z type meandering main pancreatic duct. Mean santorinicele size was 4.5 × 4.0 mm (horizontal × vertical diameters; range, 3.5–5.7 × 3.4–5.3 mm). Two of the five patients with SWOPP had undergone ERCP but no santorinicele was detected on ERCP in either patient.

**Table 1:** Features of santorinicele without pancreas divisum pathophysiology

**Case, group**	**Age, gender**	**Chief complaint (diagnosis)**	**History**	**Family history**	**Alcohol consumption (g/day)**	**Brinkman index**	**Ductal anomaly**	**Size**^**3 **^**(mm)**
1, P	67, F	Asymptomatic (Osler-Rendu-Weber disease)	CP^1^	Gastric cancer	N/A	N/A	-	3.5 × 3.5
2, P	72, F	Abdominal pain (gall stones and cholangitis)	CP^1^, alcoholic hepatitis, nephrosclerosis	-	150	150	MMPD	3.8 × 3.8
3, P	67, M	Jaundice (suspected of cholangiocarcinoma)	Post-ERCP acute pancreatitis^2^	-	50	920	-	5.3 × 5.3
4, P	77, F	Abdominal pain (CP^1^)	CP^1^, adenomyomatosis	-	0	0	-	5.7 × 4.2
5, P	85, M	Back pain (colon diverticulosis)	N/A	-	0	0	MMPD	4.2 × 3.4

^1^Diagnosed according to the latest diagnostic criteria for chronic pancreatitis [15] using magnetic resonance imaging, computed tomography, endoscopic retrograde cholangiopancreatography, and ultrasonography; ^2^Diagnosed according to the latest diagnostic criteria for acute pancreatitis [14]; ^3^Size of santorinicele (horizontal diameter × vertical diameter); CP, chronic pancreatitis; ERCP, endoscopic retrograde cholangiopancreatography; MMPD, meandering main pancreatic duct (reverse-Z type) [17]; N/A, not available; P, Patient group.

### Clinical and radiographic features of SWPD

The clinical and radiographic features of patients with SWPD are summarized in Table 2. Four of six patients (67%) were asymptomatic and the other two (33%) had abdominal pain due to biliary disease. None had a history of pancreatitis. Mean alcohol consumption was 10.8 g/day (range, 0–20 g/day). Mean Brinkman index was 2 (range, 0–12.5). No case of SWPD was accompanied by peri-ampullary diverticulum or any pancreaticobiliary anomaly except for pancreas divisum. Mean santorinicele size was 6.9 × 5.3 mm (horizontal × vertical diameters; range, 5.5–9.9 × 3.8–8.3 mm). None of the six patients had undergone ERCP.

**Table 2:** Features of santorinicele with pancreas divisum

**Case, group**	**Age, gender**	**Chief complaint (diagnosis)**	**History**	**Family history**	**Alcohol consumption (g/day)**	**BI**	**Ductal anomaly**	**Size**^**1 **^**(mm)**
6, P	75, M	Asymptomatic (adenomyomatosis)	DM, RF	DM, hepatitis,	17	0	PDAVD	6.5 × 4.2
7, P	56, F	Asymptomatic (AVM^2^)	CI	LC, Parkinson disease	0	0	Classical PD	5.5 × 3.8
8, P	76, M	Epigastralgia (gallstone)	CI, Ischemic colitis	Ileus	18	0	Classical PD	6.7 × 5.3
9, P	56, M	Hypochondrial pain (cholangitis)	Asthma, DM, hepatic cancer, rectal cancer	Hepatitis	20	0	PDAVD	6.7 × 5.3
10, P	78, M	Asymptomatic (tiny pancreatic cyst)	Hepatitis	RF, TB	10	0	Incomplete PD	5.9 × 4.7
11, C	74, M	Asymptomatic (negative study)	Hypothyroidism, RF	N/A	0	12.5	PDAVD	9.9 × 8.3

^1^Size of santorinicele (horizontal diameter × vertical diameter); ^2^Multiple pulmonary lesions but not confirmed as Osler-Rendu-Weber syndrome; AVM, arteriovenous malformation; BI, Brinkman index; C, Community group; CI, cerebral infarction; CP, chronic pancreatitis; DM, type 2 diabetes mellitus; ERCP, endoscopic retrograde cholangiopancreatography; LC, liver cirrhosis; N/A, not available; P, Patient group; PD, pancreas divisum; PDAVD, pancreas divisum with absent ventral duct; RF, renal failure; TB, tuberculosis.

### Statistical results

The incidence of SWOPP was 5/2035 (0.25%; 95% confidence interval, 0.08–0.57%) in the Patient group and 0/2905 (0.00%; 95% confidence interval, 0.00–0.10%) in the Community group. Fisher's exact test revealed that the prevalence of SWOPP was significantly higher in the Patient group than in the Community group (P = 0.01). The incidence of pancreatitis and chronic pancreatitis was significantly higher in patients with SWOPP than in the Patient group (pancreatitis, 11.5% vs. 80%, P = 0.01, odds ratio = 6.9; chronic pancreatitis, 8.50% vs. 60%, P = 0.02, odds ratio = 7.0) and in the Community group (pancreatitis, 1.10% vs. 80%, P < 0.001, odds ratio = 65; chronic pancreatitis, 0.41% vs. 60%, P < 0.001, odds ratio = 120). Patients with SWOPP were older than those in the Patient group [no statistical significance after family-wise error correction (Student’s *t*-test, P = 0.047)]. Subjects in the Patient group were older than those in the Community group (Student’s *t*-test, P < 0.01). No other significant difference was detected among patients with SWOPP, the Patient group, and the Community group in terms of gender, alcohol consumption, or Brinkman index.

The incidence of SWPD was 5/2035 (0.25%; 95% confidence interval, 0.08–0.57%) in the Patient group and 1/2905 (0.03%; 95% confidence interval, 0.001–0.19%) in the Community group. No significant difference was detected regarding the prevalence of SWPD between the Patient and Community groups (Fisher’s exact test, P = 0.09). No significant difference was detected regarding age between subjects with SWPD and the Patient group (Student’s *t*-test, P = 0.27). No other significant difference was detected among patients with SWPD, the Patient group, and the Community group in terms of gender, alcohol consumption, or Brinkman index.

No statistical significance was detected between the incidence of SWOPP and that of SWPD in either group (P = 1.00 for either group). Santorinicele size was significantly larger in subjects with SWPD than in those with SWOPP (P = 0.02).

## Discussion

In the present cross-sectional study, we first determined the gross prevalence of SWOPP and SWPD in both patient and community populations, based on 1.5/3 T high-field MR studies. The results showed SWOPP to be a relatively rare condition closely associated with a history of pancreatitis. Our series appears to include the second to sixth reported cases of SWOPP.

With one exception, all previously reported cases of santorinicele have been in patients with pancreas divisum, either complete or incomplete [2-7], or in patients with dominant dorsal duct [12,13], which has a similar pathophysiology to pancreas divisum [18]. The exception was a case of SWOPP accompanied by bifid tail of the pancreas [11], which is a congenital anomaly where the primitive pancreatic channels fail to fuse into a single main duct during development of the pancreas.

Of the various morphological patterns reported of the dorsal pancreatic duct [8-10], the saccular and spindle types may appear similar to santorinicele in shape; however, their pathophysiologies are quite different. For example, patency of the dorsal pancreatic duct was 14% in saccular type and 93% in spindle type [8-10]. This is why we defined santorinicele as a saccular formation of the terminal end of the dorsal pancreatic duct, and why the spindle type was excluded from the study. It is speculated that the inability to decompress pancreatic ducts when there is low patency of the dorsal pancreatic duct can lead to pancreatitis [9,10] and to santorinicele formation. The present results agree with those of previous reports and support the hypothesis that santorinicele is associated with the onset of pancreatitis.

The incidence of SWOPP in the present study (0.25%) was the same as that of SWPD but it was low compared with the results of a previous ERCP-based study regarding saccular-type dorsal pancreatic duct (2.3%) [9]. The reason for this discrepancy is probably because of the mixed population of santorinicele with and without pancreas divisum, modality-dependent selection bias, and the lower resolution of MRCP than ERCP in the previous study.

It is unclear whether santorinicele itself, with or without pancreas divisum, is congenital in origin or is an acquired lesion secondary to a stenosis of the dorsal duct orifice. Several studies speculated that santorinicele is a congenital anomaly [4,11,12] analogous to a choledochocele [12]. A case of santorinicele is reported in a paediatric patient in whom duodenal diverticulum was not the cause of the dorsal ductal obstruction [4]. SWPD is reported as a possible cause of the relative stenosis of the accessory papilla, which, in association with unfused dorsal and ventral ducts, results in the high intraductal pressure responsible for the recurrent episodes of acute pancreatitis [3-5,7,13]. Therefore, sphincterotomy or balloon dilatation is believed to be effective in patients with santorinicele and pancreatitis [3-5,7,13]. However, other reports speculate that it is an acquired state because most cases of SWPD are reported in elderly patients [12,13] and SWPD is associated with an adjacent duodenal diverticulum [12]. Structural changes may contribute to the acquired mucosal weakness, thereby facilitating the formation of a santorinicele [12]. Several past reports speculated that SWOPP is a rare congenital pathology rather than an acquired condition [4,11,12].

In the present case series, SWOPP was not accompanied by a major morphological abnormality of the pancreatic ducts. Although there were many more cases of pancreatitis in the MRCP-based Patient group than in the Community group, a significantly high proportion of patients with SWOPP were associated with pancreatitis and chronic pancreatitis, and these patients were older than the other subjects (not statistically significant). Although mean age was older in the Patient group than in the Community group, this finding does not appear to be in conflict with the hypothesis that SWOPP is an acquired condition that is closely related to a history of pancreatitis, especially chronic pancreatitis. Accordingly, the mechanism by which a santorinicele develops is speculated to be impeded pancreatic flow in a less patent dorsal pancreatic duct, causing the onset of pancreatitis [8-10]; however, not all patients with pancreatitis develop santorinicele and its risk factors are not fully elucidated. In addition, two of the five present cases of SWOPP had a reverse-Z type meandering main pancreatic duct, which is a common anatomical variation of the ventral pancreatic duct associated with idiopathic recurrent acute pancreatitis [17]; however, the association of SWOPP with meandering main pancreatic duct might be explained by assuming pancreatitis as a potential confounding factor.

In the present study, santorinicele size was larger in SWPD than in SWOPP, which indicates that ductal pressure at the terminal portion of the dorsal pancreatic duct may be higher in SWPD than in SWOPP. However, the present cases of SWPD were dominantly asymptomatic and not associated with pancreatitis. SWPD is speculated to predispose to pancreatitis [3], but the present results suggest that SWPD does not always induce pancreatitis. Further investigation may explain the cause of santorinicele as a variation in the connection of the ventral and dorsal pancreatic ducts, as suggested in several studies [16,17,19,20], as variable patency of the dorsal pancreatic duct [8-10], or as some unknown congenital factor such as a gene mutation.

There is a report of a single case of Wirsungocele [21], a focal saccular dilatation of the terminal portion of the ventral pancreatic duct, associated with recurrent acute pancreatitis [22]. This might be a close relative of SWOPP or SWPD; however, its pathophysiology remains unclear.

Methodologically, we used MRCP as the gold standard for depicting santorinicele because this modality is reported as highly sensitive and specific (approximately 90%–100%) in investigating pancreatic ductal anatomy [16,23,24]. We did not use ERCP as the gold standard because it is not applicable to a healthy population, the form of the terminal end of the dorsal duct can be altered via cannulation during the procedure, and its success rate is known to be low [25]. In the present study, ERCP failed to depict santorinicele. Thus, ERCP is a less appropriate modality than MRCP for assessing santorinicele. We did not use secretin-enhanced MRCP, which is reported to improve santorinicele visualization [6] but also causes abnormal responses of the main pancreatic duct to secretory stimulation [26], because it is unavailable in our country. Accordingly, we may have underestimated the rate of SWOPP in the present study. A further limitation is that we did not perform histopathological investigation of all detected santoriniceles, for the reason that this was not clinically indicated.

In clinical practice, santorinicele is more easily diagnosed with MRCP than with ERCP. The present results show that santorinicele may not be associated with pancreas divisum, and appears to be caused by impeded pancreatic flow in the dorsal pancreatic duct. Sphincterotomy or balloon dilatation may be effective in patients with intractable pancreatitis with SWOPP, as well as in those with SWPD.

## Conclusion

We presented the second to sixth reported cases of SWOPP and the first assessment of the gross prevalence of this relatively rare condition in both patient and community populations, proving that Santorinicele is not always accompanied by pancreas divisum. We also established that SWOPP is found mostly in patients suffering pancreatitis, especially chronic pancreatitis, and we consider that it is an acquired pathology.

## Competing interests

The authors declare that they have no competing interests.

## Authors’ contributions

WG drafted the manuscript and conceived the study concept and design. HA and KH performed analysis and data interpretation. MA, MT, SK, and NO participated in critical revision of the manuscript for important intellectual content. NH, EM, TY, and KU performed acquisition of data. KK and KO gave final approval of the version to be published. All authors read and approved the final manuscript.

## Pre-publication history

The pre-publication history for this paper can be accessed here:

http://www.biomedcentral.com/1471-230X/13/62/prepub
